# Examining the mediating effects of motivation between job insecurity and innovative behavior using a variable-centered and a person-centered approach

**DOI:** 10.3389/fpsyg.2023.1284042

**Published:** 2023-11-30

**Authors:** Bing Ma, Yarong Zhou, Hermann Lassleben, Guimei Ma, Rong Yang

**Affiliations:** ^1^School of Management, Xi'an Polytechnic University, Xi'an, Shaanxi, China; ^2^ESB Business School, Reutlingen University, Reutlingen, Baden-Württemberg, Germany

**Keywords:** job insecurity, innovative behavior, intrinsic motivation, impression management motivation, variable-centered and person-centered approach

## Abstract

**Introduction:**

The fierce market competition environment makes employees feel insecure at work. While it is difficult for enterprises to provide employees with a sense of security, they have to rely on employees’ innovative behavior to seek competitive advantage. Therefore, this study focuses on how employees engage in innovative behavior when they face job insecurity.

**Methods:**

Using a variable-centered approach, this study aims to examine the mediating effects of intrinsic and impression management motivation in the relationship between quantitative and qualitative job insecurity and innovative behavior, including proactive and reactive innovative behavior. In addition, a person-centered approach is used to investigate whether it is possible to distinguish different combinations of quantitative and qualitative job insecurity, and examine the effect of these job insecurity profiles on motivation and innovative behavior. We used 503 data sets collected via the Credamo platform in China into the data analysis.

**Results:**

The study found that quantitative job insecurity affects proactive and reactive innovative behavior through impression management motivation and that qualitative job insecurity affects proactive and reactive innovative behavior through intrinsic and impression management motivation. In addition, three job insecurity profiles were identified: balanced high job insecurity, balanced low job insecurity, and a profile dominated by high quantitative job insecurity, all of which have significantly different effects on motivation and innovative behavior.

**Discussion:**

This study contributes to provide new insights into the relationship between job insecurity and innovative behavior and compensate for the limitation of the traditional variable-centered approach that cannot capture heterogeneity within the workforce.

## Introduction

Innovation is critical to organizational success, so organizations must rely on employee innovative behavior to achieve organizational innovation (Sternberg and Shoham, 2022), even if environmental changes (i.e., the development of artificial intelligence and the advent of the post-pandemic era) prevent them from providing job security for employees (Lin et al., 2021; Yam et al., 2023). Employee innovative behavior is defined as any individual behavior that generates, introduces, or applies beneficial novelty at any organizational level (Kleysen and Street, 2001). Previous studies have mainly considered innovative behavior as a voluntary behavior in which employees actively generate new ideas and seek support and practice (Scott and Bruce, 1994). In fact, employees may also involuntarily engage in reactive innovative behavior due to pressures from the organizational environment. Thus, innovative behavior can be distinguished into proactive and reactive innovative behavior, and the latter should also not be ignored in research (Zhao et al., 2015).

Although previous studies have discussed the effect of job insecurity on innovative behavior (Teng et al., 2019; Wang et al., 2019; Jiang et al., 2022), little is known about the mechanisms involved. To date, research has mainly tested individual emotional or attitudinal reactions (e.g., job engagement, psychological contract violation) as mediators (De Spiegelaere et al., 2014; Niesen et al., 2018). In our view, the impact of job insecurity on innovative behavior can be better understood by including motivation to innovate as an additional variable in the research design. Employees engage in innovative behavior not only out of self-interest, but also because they perceive opportunities to make a positive impression on managers or peers (Morrison and Bies, 1991). On the one hand, innovative behavior helps to improve performance, attract the attention of supervisors, or receive recognition, so engaging in it can be used to improve one’s social image. On the other hand, employees who engage in innovative behavior, which is a type of extra-role behavior that is beneficial to the organization, are more likely to be recognized by the organization. Thus, we consider both intrinsic and impression management motivations as mediators between job insecurity and innovative behavior.

In addition, job insecurity can be divided into two dimensions: quantitative job insecurity (concerns about losing current job) and qualitative job insecurity (concerns about losing valued job characteristics such as salary increases or development opportunities) (Hellgren et al., 1999). Previous studies on job insecurity have mainly adopted a variable-centered approach, ignoring the complex situation that individuals may perceive both quantitative and qualitative job insecurity at the same time. Debus et al. (2020) called on researchers to pay attention to how perceived quantitative and qualitative job insecurity are combined in the individual to form an overall effect, which cannot be solved by a variable-centered approach. Therefore, it is necessary to complement it with a person-centered approach to make the results more realistic.

To address these research gaps, we follow the logical process of “perception-motive-response” and examine the relationship between job insecurity, motivation to innovate, and innovative behavior. The originality and value of our study is twofold: (1) Using a variable-centered approach, we examine the mediating roles of intrinsic and impression management motivation between job insecurity and innovative behavior, including quantitative and qualitative job insecurity, proactive and reactive innovative behavior. In this way, we hope to add new insights to the literature by including reactive innovative behavior as an outcome variable and by opening the black box of the relationship between job insecurity and innovative behavior by including individual motives as mediators. (2) Using a person-centered approach, we explore the potential profiles of job insecurity and their impact on motivation and innovative behavior, aiming to compensate for the limitation of the variable-centered approach, which cannot capture heterogeneity within the workforce.

As China provides a suitable environment for researching the effect of job insecurity on innovative behavior issues, we conduct this research in China. On the one hand, many Chinese employees are currently facing job insecurity, not only risk of unemployment, but also concerns about job quality and prospects. Although the National Bureau of Statistics of China revealed the average urban unemployment rate in 2022 was 5.6% (National Bureau of Statistics of the People’s Republic of China, 2023), many employees still worry about their job continuity due to the introduction of new technologies and the aftermath of the pandemic. Moreover, the 16th China (CIIC) EAP Annual Conference Report revealed that job development issues, such as promotion, are important sources of employees’ perceived work pressure (CIIC Occupational Mental Health Center, 2019). On the other hand, as the Chinese government has been encouraging companies and employees to innovate in recent years, employees may engage in innovative behavior voluntarily or involuntarily. Sometimes, when companies set policies and requirements related to innovation, employees may be forced to engage in reactive innovative behavior that is inconsistent with their own cognitions.

## Theoretical background and hypotheses development

### Job insecurity and intrinsic motivation

Intrinsic motivation is the motivation of individuals to engage in activities because they find them interesting and enjoyable (Amabile et al., 1996). According to self-determination theory (SDT), the generation of intrinsic motivation and the internalization of extrinsic motivation can be promoted by satisfying the three basic psychological needs of competence, autonomy, and belongingness (Deci and Ryan, 1980). Job insecurity frustrates the three basic psychological needs, leading to a decrease in intrinsic motivation.

Employees facing high levels of quantitative job insecurity may lose their sense of control to master the environment and achieve desired outcomes, resulting in a sense of helplessness, counteracting their competence needs (Vander Elst et al., 2012). In addition, the perceived risk of job loss may affect employees’ sense of freedom of choice and decision making, thwarting their autonomy needs (Vander Elst et al., 2012). Finally, quantitative job insecurity implies the risk of unemployment, which leads to the loss of relationships with colleagues and social identity as an employee (Ma et al., 2016; Selenko et al., 2017), threatening employees’ belongingness needs. Taken together, high levels of quantitative job insecurity are expected to lead to a decrease in employees’ intrinsic motivation due to the frustration of their basic psychological needs.

Qualitative job insecurity involves the fear of losing important job characteristics, which means that the nature of tasks and working conditions may change in the future. Employees who experience high levels of qualitative job insecurity may feel pessimistic about the future prospects of the organization and their own career development opportunities within it (Yang et al., 2019). They may worry about the competencies required for future jobs and whether their competencies are sufficient to achieve future goals, which inhibits their competency needs. In addition, when employees perceive that important job characteristics (e.g., promotion) are threatened, they often feel powerless and lack a sense of control (Vander Elst et al., 2014), which may frustrate their autonomy needs. Finally, perceptions of a damaged employment relationship and concerns about poor career development opportunities may lead employees to lack a sense of belonging, which may frustrate their belongingness needs (Vander Elst et al., 2014). Taken together, it can be assumed that employees experiencing high levels of qualitative job insecurity may suffer from a decline in intrinsic motivation due to the frustration of basic psychological needs.

Using a sample of 152 researchers from a South Korean manufacturing company, Shin et al. (2019) found a negative relationship between job insecurity and intrinsic motivation. Previous research also suggests that in the context of high job insecurity, environmental instability causes employees to pay more attention to the risks of innovative activities in terms of rewards and punishments (Zhou and Long, 2011). That is, the innovative behavior generated by extrinsic motivation will have a crowding out effect on intrinsic motivation. Therefore, we assume that both quantitative and qualitative job insecurity will reduce employees’ intrinsic motivation to engage in innovative behavior, and propose hypotheses H1a and H1b:

*H1*a: Quantitative job insecurity has a negative effect on intrinsic motivation.

*H1*b: Qualitative job insecurity has a negative effect on intrinsic motivation.

### Job insecurity and impression management motivation

Impression management motivation refers to the tendency of individuals to try to influence the image that others have of them (Rosenfeld et al., 1995, 2001). In general, people seek to be viewed positively by others or to avoid being viewed negatively. In this study, we focus on the former. Because job insecurity refers to events or threats that have not yet occurred, it is likely to spur job preservation motivation and encourage employees to portray themselves as great contributors in order to prevent the loss of their jobs or important features of their jobs (Shoss, 2017; Shoss et al., 2023). As a result, employees experiencing job insecurity may be motivated to engage in impression management in order to influence actual outcomes.

As quantitative job insecurity involves the risk of unemployment, it threatens employees’ security needs (Long et al., 2022). As a result, employees may focus on their jobs, believing that this is the best way to manage the risk and avoid unemployment. Especially in traditional Chinese culture, jobs are very important to individuals. For the Chinese, unemployment is not only a loss of security, but also a loss of face because their value is not recognized. Because Chinese culture also promotes a spirit of struggle and perseverance, employees are less likely to break the pot and more likely to feel ashamed and then be brave. If employees experiencing quantitative job insecurity feel that creating a positive image at work will help them retain their jobs, they may be motivated to engage in impression management and take actions to demonstrate their value to the organization, leaders, and coworkers (Shoss and Probst, 2012; Shoss, 2017). In fact, previous research has also shown that job insecurity can lead employees to engage in upward impression management (Huang et al., 2013).

Since qualitative job insecurity involves the risk of losing important job characteristics, it mainly threatens employees’ growth needs (Long et al., 2022). Employees who experience high levels of qualitative job insecurity may become pessimistic about the future of the organization and their career development opportunities within the organization, which negatively affects their job preservation motivation (Yang et al., 2019). Previous research found that employees facing qualitative job insecurity have low job involvement, do not fear being laid off, and have relatively low job preservation motivation (Tu et al., 2020). Obviously, development prospects in the organization are key to job preservation motivation. Because qualitative job insecurity prevents employees from finding meaning in their jobs, they may more easily decide to leave (Tu et al., 2020) and seek better opportunities elsewhere rather than engage in impression management with their current employer.

Therefore, we expect that quantitative job insecurity will increase impression management motivation, while qualitative job insecurity will decrease it, and propose hypotheses H2a and H2b:

*H2*a: Quantitative job insecurity has a positive effect on impression management motivation.

*H2*b: Qualitative job insecurity has a negative effect on impression management motivation.

### The mediating role of intrinsic motivation

Proactive innovative behavior is a set of behaviors that employees engage in on their own to promote environmental improvement or self-improvement (Frese et al., 1996). Based on an individual’s needs, interests, and efforts, intrinsic motivation is considered the driving force behind proactive innovative behavior. The higher their intrinsic motivation, the more likely people are to engage in somewhat risky activities where the outcome is uncertain. Previous research has also found that intrinsic motivation has a positive effect on proactive innovative behavior (Montani et al., 2021). Specifically, individuals with high intrinsic motivation are more likely to seek and obtain important information, generate and implement new ideas, and try to solve problems by taking different perspectives (Montani et al., 2021).

Reactive innovative behavior, on the other hand, is a more passive behavior that employees engage in, often driven by extrinsic motivation (Zhao et al., 2015). In this case, employees force themselves to innovate under the pressure of the organizational environment, ultimately against their own beliefs. Individuals with low intrinsic motivation have little interest in their work and little sense of purpose. They also lack the enthusiasm and autonomy to be proactive innovators. However, they are usually focused on their work and receptive to external incentives (Zhou and Long, 2011), which favors reactive innovative behavior. They are more likely to be influenced by extrinsic motivators, such as organizational rewards for innovation. Even if they are not convinced of the innovations, they implement them, but rather passively. Therefore, we propose hypotheses H3a and H3b.

*H3*a: Intrinsic motivation has a positive effect on employees’ proactive innovative behavior.

*H3*b: Intrinsic motivation has a negative effect on employees’ reactive innovative behavior.

According to SDT, employees are more likely to develop intrinsic motivation the more their work environment allows them to satisfy their needs for competence, autonomy, and belongingness (Flannery, 2017). Job insecurity is a threat that puts employees under stress (De Witte et al., 2016; Ma et al., 2019). In the case of quantitative job insecurity, it is the threat of unemployment; in the case of qualitative job insecurity, it is the threat of losing valuable job characteristics. In both cases, the needs for competence, autonomy, and belongingness are frustrated, leading to a decrease in intrinsic motivation and affecting innovative behavior. As discussed above, we expect intrinsic motivation to promote proactive innovative behavior while inhibiting reactive innovative behavior. Combined, we therefore hypothesize that both quantitative and qualitative job insecurity will have a negative effect on proactive innovative behavior and a positive effect on reactive innovative behavior due to their negative effect on intrinsic motivation. Therefore, we propose hypotheses H4a and H4b:

*H4*a: Intrinsic motivation plays a mediating role between quantitative job insecurity and proactive and reactive innovative behavior.

*H4*b: Intrinsic motivation plays a mediating role between qualitative job insecurity and proactive and reactive innovative behavior.

### The mediating role of impression management motivation

Impression management can be seen as a form of active self-management aimed at improving one’s image and gaining recognition from others. Based on the assumption that it is human nature to seek the recognition of others (Fülöp et al., 2023), it stands to reason that employees, driven by impression management motivation, tend to use innovative behavior as a strategy to influence others. They participate in innovation to improve their image and gain recognition. Previous research has shown that employees who strive to develop a positive image and make a good impression are more likely to engage in innovative behaviors (Zhao and Zhao, 2019). Therefore, we expect that impression management motivation encourages employees to engage in proactive innovative behaviors.

As a manifestation of extrinsic motivation, impression management motivation can also encourage employees to engage in reactive innovative behavior. In China, a wide range of policy-driven innovations have emerged in organizations in recent years, setting innovation goals for employees and encouraging them to innovate (Yang et al., 2020). Even if these innovations are not in line with their own beliefs, employees, especially those with high impression management motivation, will still engage in innovative behavior, but more reactively, in order to make a good impression on their managers and peers. Therefore, we propose hypotheses H5a and H5b:

*H5*a: Impression management motivation has a positive effect on employees’ proactive innovative behavior.

*H5*b: Impression management motivation has a positive effect on employees’ reactive innovative behavior.

Job insecurity is a threat that has not yet materialized. Depending on their job preservation motivation, employees may engage in different behaviors to cope with this threat (Shoss, 2017). For example, they may engage in innovative behaviors in the expectation that they can avert the threat by increasing their esteem by others, thereby securing their jobs.

Employees facing quantitative job insecurity will focus on their jobs. The effort to transform insecurity into security drives their job preservation motivation (Shoss, 2017). The more pronounced the quantitative job insecurity, the more likely it is that employees will also develop impression management motivation. They will try to improve their image by exhibiting the innovative behavior expected by the organization in order to demonstrate their value and reduce the risk of job loss (Mustafa and Ramos, 2018). In this regard, high quantitative job insecurity can lead to impression management motivation, which in turn promotes the development of proactive and reactive innovative behaviors.

Employees facing qualitative job insecurity are concerned about the future of their organization and, in particular, about their own career development prospects. Because they are pessimistic about the future of the organization and their own career development prospects within it (Long et al., 2022), they are open to development opportunities outside their current organization. In the absence of job preservation motivation, they are also less likely to engage in impression management. The more pronounced the qualitative job insecurity, the less likely it is that the desired return (e.g., salary increase, etc.) will be achieved, even if a good image is built through innovative behavior. Consequently, we expect that the higher the level of qualitative job insecurity, the lower the impression management motivation, which inhibits both proactive and reactive innovative behavior. Therefore, we propose hypotheses H6a and H6b:

*H6*a: Impression management motivation plays a mediating role between quantitative job insecurity and proactive and reactive innovative behavior.

*H6*b: Impression management motivation plays a mediating role between qualitative job insecurity and proactive and reactive innovative behavior.

### Differential effects of job insecurity profiles on employees’ motivation and innovative behavior

The distinction between quantitative and qualitative job insecurity is primarily theoretical and focuses on different aspects of job insecurity (Piccoli et al., 2017; Tu et al., 2020). From an empirical perspective, employees usually face a complex threat situation in which different degrees of qualitative and quantitative job insecurity coexist (Fülöp et al., 2022). Therefore, job insecurity may not only vary overall or at the level of dimensions, but also with respect to the combinations of the respective levels of the dimensions. Previous studies have mainly used a variable-centered approach, treating each variable as a separate entity. Combinations have been neglected. In order to examine whether the combined experience of quantitative and qualitative job insecurity leads to specific outcomes, profiles must be created. Therefore, this study creates and examines personal profiles to determine in which combinations quantitative and qualitative job insecurity typically occur and with what effects. This compensates for the aforementioned shortcomings of the variable-centered approach.

**Figure 1:**
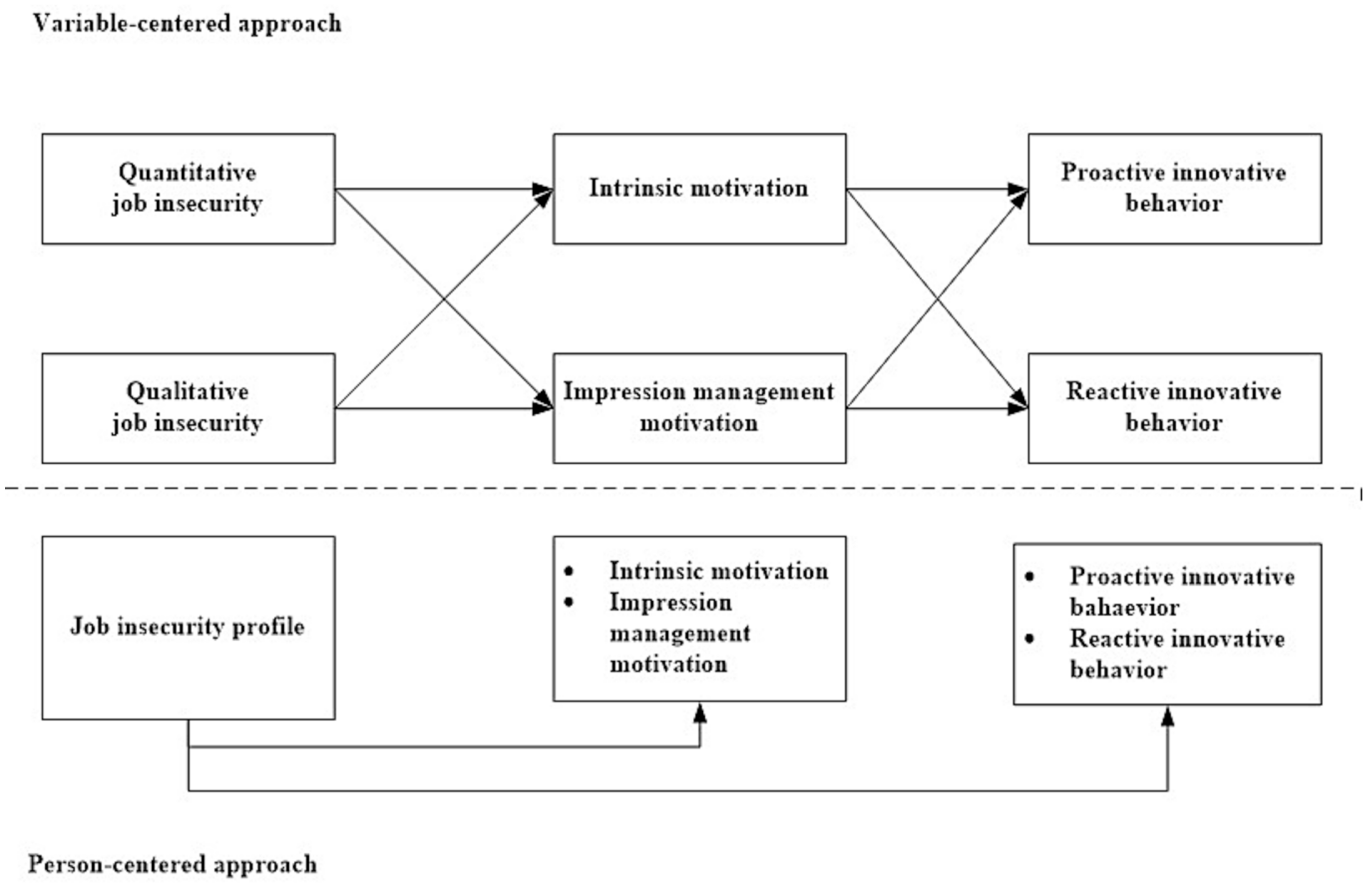
The conceptual model.

For this purpose, we use the method of latent profile analysis. In this method, individuals are classified into groups (profiles) based on empirical differences in combinations of quantitative and qualitative job insecurity and their similarities and differences in terms of motivation and innovative behavior. Since this person-centered approach is inductive in nature and the number and characteristics of the profiles cannot be predicted in advance, we use a research question rather than hypotheses to guide the empirical analysis. In sum, the research model is shown in Figure 1.

RQ: Which profiles can be identified with regard to the combination of quantitative and qualitative job insecurity and what are their consequences for employees’ motivation and innovative behavior?

## Method

### Samples and procedures

Assuming that innovative behavior is of a general nature and includes groundbreaking innovations as well as small, everyday improvements that are equally important for the success of a company, data was collected from Chinese employees in all types of jobs and companies using the online platform Credamo. The platform allows researchers to control who can participate in a study and monitor the completion time. In this way, the questionnaire can be sent to employees for paid administration using precise push functions. To ensure the quality of the dataset, we included two attention check questions, with the platform automatically rejecting participants who did not select the correct answer.

Data collection and processing were conducted in full compliance with ethical guidelines, and participants were informed that their identity would not be disclosed. The questionnaire was preceded by a description of the purpose of the study. The survey instrument consisted of two parts: (a) demographic variables and (b) measurement of the dependent, independent and mediator variables of the research design on 7-point Likert scales. A total of 610 questionnaires were collected in November 2021. After eliminating unrealistically short completion times, repeated participation and regular and extreme responses, 503 valid data sets were obtained, corresponding to a response rate of 82.46%. In the final sample, 55% of the participants were women and 45% were men. In terms of age, the 21–30 age group predominated with 55%. The educational level of the participants was predominantly undergraduate with 73%, followed by postgraduate with 13%. Table 1 shows the demographics of the sample.

**Table 1:** Demographics of respondents.

Demographic Variables	Frequency (*N* = 503)	Percentage (%)
*Gender*		
Male	224	44.5
Female	279	55.5
*Age*		
Under 20 years	7	1.4
21–30 years	275	54.7
31–40 years	189	37.6
41–50 years	26	5.2
Over 51 years	6	1.2
*Education*		
High school and below	20	4.0
Associate degree	47	9.3
College degree	369	73.4
Graduate degree or above	67	13.3
*Tenure*		
<6 months	20	4.0
6 months ≤ Tenure<1 year	18	3.6
1 year ≤ Tenure<3 years	98	19.5
3 years ≤ Tenure<7 years	234	46.5
7 years ≤ Tenure<10 years	80	15.9
≥10 years	53	10.5
*Job type*		
R&D/Technology	207	41.2
Marketing	88	17.5
Production/Process/Quality	75	14.9
HR/Administration/Finance	111	22.1
Operation/Logistics	16	3.2
Other	6	1.2

### Measures

All measures were adopted from the literature and administered in Chinese. Except for the demographic variables, participants were asked to respond on 7-point Likert scales ranging from 1 (strongly disagree) to 7 (strongly agree).

Job insecurity was measured using a seven-item scale developed by Hellgren et al. (1999). Quantitative job insecurity was measured with three items. A sample item reads: “I feel unsafe about losing my job.” Qualitative job insecurity was measured with four reverse-coded items. A sample item reads: “My future career opportunities in the organization are feasible.”

Intrinsic motivation was measured by a three-item subscale of the multidimensional work motivation scale developed by Gagné et al. (2015). To make the measure more suitable for research, we added an innovation scenario to the item description. A sample item reads: “I set innovation goals and work hard for them because work makes me happy.”

Impression management motivation was measured using Zhao and Zhao’s (2019) scale, which is in Chinese language and applicable to the Chinese innovation scenario. The scale consists of seven items to measure people’s tendency to be viewed positively by others. A sample item reads: “I try to put forward new ideas in my work to make my leaders or colleagues think I am creative.”

Proactive innovative behavior was measured using Scott and Bruce’s (1994) six-item scale. A sample item reads: “I often generate some creative ideas.”

Reactive innovative behavior was measured using a scale by Yang et al. (2020). The scale consists of six items adapted to the research context of this study. A sample item reads: “I do not need to go all out or surpass myself when I innovate, just need to meet the innovation requirements.”

Consistent with previous studies, five demographic variables were used as control variables: gender, age, education, tenure, and job type.

## Results

### Common method bias

According to Harman (1976), a single factor accounting for more than 50% of the variance indicates common method bias. The first factor accounted for 33.13% of the variance, indicating that there was no apparent common method bias in the study.

### Measurement model

Confirmatory factor analysis was used to evaluate the measurement model and to test convergent and discriminant validity. Cronbach’s alpha was found to be >0.8, AVE > 0.5, and CR > 0.8, meeting standard requirements (Hair et al., 1998; Ringle et al., 2020; see Table 2).

**Table 2:** Validity and reliability of the latent variable constructs in the measurement model.

Constructs	Item	Standardized factor loading	Cronbach’s alpha	CR	AVE
Quantitative job insecurity	JI1	0.876	0.864	0.868	0.687
	JI2	0.771			
	JI3	0.837			
Qualitative job insecurity	JI4	0.807	0.858	0.863	0.613
	JI5	0.783			
	JI6	0.703			
	JI7	0.832			
Intrinsic motivation	IM1	0.864	0.871	0.873	0.697
	IM2	0.803			
	IM3	0.836			
Impression management motivation	IMM1	0.796	0.912	0.913	0.601
	IMM2	0.808			
	IMM3	0.774			
	IMM4	0.807			
	IMM5	0.779			
	IMM6	0.725			
	IMM7	0.734			
Proactive innovative behavior	PIB1	0.786	0.877	0.878	0.546
	PIB2	0.770			
	PIB3	0.698			
	PIB4	0.672			
	PIB5	0.687			
	PIB6	0.809			
Reactive innovative behavior	RIB1	0.876	0.888	0.893	0.590
	RIB2	0.748			
	RIB3	0.843			
	RIB4	0.873			
	RIB5	0.690			
	RIB6	0.512			

To measure discriminant validity, we evaluated the square root of the AVE. If the square root of the AVE is higher than the correlation between the structures, it indicates that the discriminant validity is good (Fornell and Larcker, 1981). Table 3 shows that the data meets the requirements of standards.

**Table 3:** Discriminant validity: AVE-SV comparison.

	1	2	3	4	5	6
1. Quantitative job insecurity	0.83					
2. Qualitative job insecurity	0.39^***^	0.78				
3. Intrinsic motivation	0.03	−0.37^***^	0.83			
4. Impression management motivation	0.12^**^	−0.16^***^	0.03	0.78		
5. Proactive innovative behavior	−0.01	−0.14^**^	0.54^***^	0.29^***^	0.74	
6. Reactive innovative behavior	0.21^***^	0.10^*^	−0.14^**^	0.31^***^	−0.07	0.77

Discriminant validity: AVE-SV comparison (based on Fornell and Larcker’s criteria).

***p < 0.001, **p < 0.01, *p < 0.05.

According to Kline (1998), a good model fit is indicated by the following: The Tucker-Lewis Index (TLI) and the Comparative Fit Index (CFI) are close to 1.00, while the Root Mean Square Error of Approximation (RMSEA) is equal to or less than 0.08. Regarding the measurement model, the model fit indices support an adequate fit between the model and the data given the threshold values (Hair et al., 2014) (*χ*^2^(*n* = 503) =793.05, *χ*^2^/df = 2.19, IFI = 0.95, CFI = 0.95, TLI = 0.95, and RMSEA = 0.05). Thus, the measurement model fits the data well.

### Structural model

**Figure 2:**
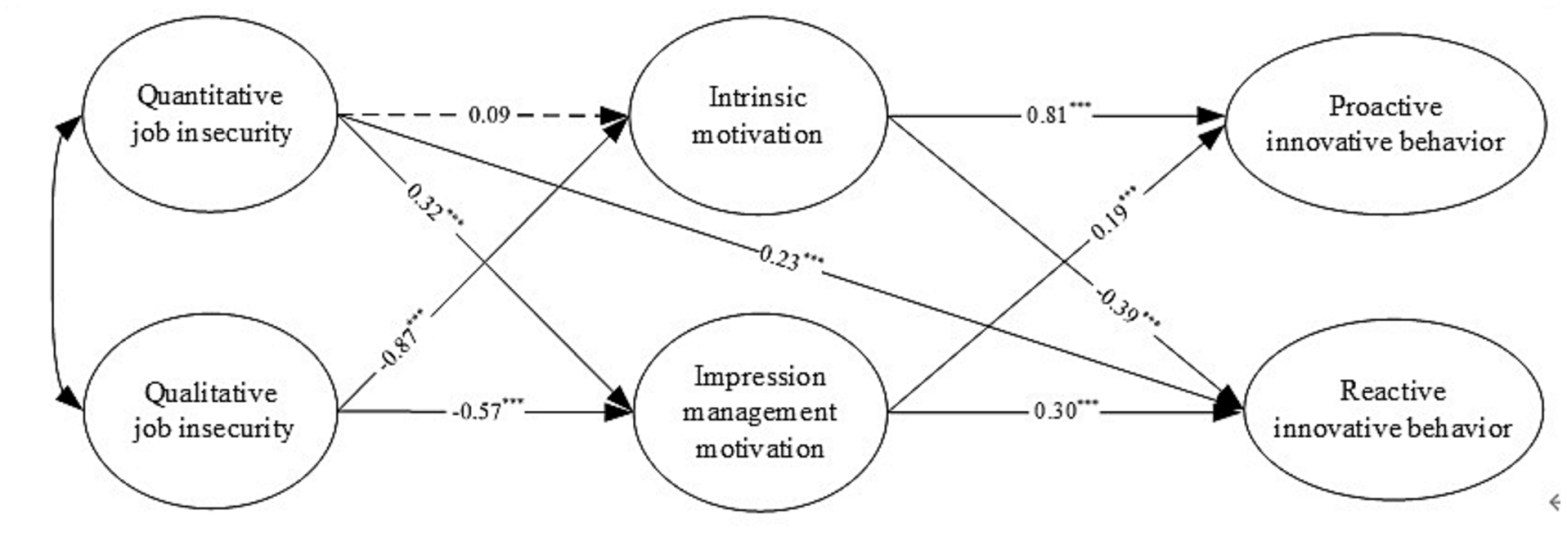
Path analysis.

AMOS 24.0 was used to test the structural model and verify the hypothesized paths. The structural model fits the data well (*χ*^2^(*n* = 503) = 802.85, *p* < 0.001, *χ*^2^/df = 2.19, CFI = 0.95, TLI = 0.95 and RMSEA = 0.05). The path analysis (see Figure 2) shows that quantitative job insecurity has no significant effect on intrinsic motivation (*β* = 0.09, *p* = 0.06, ns), but a significant positive effect on impression management motivation (*β* = 0.32, *p* < 0.001), supporting H2a but not H1a. Qualitative job insecurity is negatively correlated with intrinsic motivation and impression management motivation (*β* = −0.87, *p* < 0.001; *β* = −0.57, *p* < 0.001), supporting both H1b and H2b. Intrinsic motivation is positively correlated with proactive innovative behavior (*β* = 0.81, *p* < 0.01) and negatively correlated with reactive innovative behavior (*β* = −0.39, *p* < 0.001), supporting H3a and H3b. Impression management motivation has a significant positive effect on proactive and reactive innovative behavior (*β* = 0.19, *p* < 0.001; *β* = 0.30, *p* < 0.001), supporting H5a and H5b.

The mediating effect of intrinsic and impression management motivation between job insecurity and innovative behavior was tested using the bootstrapping method with random resampling set to 5,000. Since the results of the direct effect test showed that the relationship between quantitative job insecurity and intrinsic motivation was not significant, H4a was not tested in this study. It was found that qualitative job insecurity has a significant effect on proactive and reactive innovative behavior through intrinsic motivation (*β* = −0.58, 95% CI = [−0.71, −0.46]; *β* = 0.47, 95% CI = [0.32, 0.64]) and a significant negative effect on proactive and reactive innovative behavior through impression management motivation (*β* = −0.09, 95% CI = [−0.16, −0.05]; *β* = −0.23, 95% CI = [−0.33, −0.16]), supporting H4b and H6b. Quantitative job insecurity has a positive effect on proactive and reactive innovative behavior through impression management motivation (β = 0.04, 95% CI = [0.02, 0.06]; *β* = 0.10, 95% CI = [0.06, 0.14]). Therefore, H6a is supported.

### Latent profile analysis

#### Profiles of quantitative and qualitative job insecurity

Latent profile analysis was performed using Mplus 8.0 to identify profiles of quantitative and qualitative job insecurity. All data were processed after normalization, and latent profile analysis was performed for one to five class solutions. Following Nylund et al. (2007), we chose Bayesian Information Criteria (BIC), Likelihood Ratio Test (LMR), Bootstrap Likelihood Ratio Test (BLRT), and Entropy as indicators of fit. The BIC allows us to compare models with different numbers of classes. The lower the value, the better the BIC. LMR and BLMR both provide a value of p reference that indicates whether adding a profile improves the model fit. Entropy represents the degree of confidence that an individual is in the correct class, with a higher value (values ranging from 0 to 1) representing clearer class separation. Entropy values up to 0.40, 0.60, and 0.80 represent low, medium, and high separation, respectively.

Table 4 shows the results. The *p*-values of LMR and BLMR of Model 2 and Model 3 are significant. Compared to Model 2, Model 3 has lower AIC, BIC, ABIC and higher Entropy (greater than 0.8). Thus, Model 3 is the optimal model, which means that the three profiles provide the best fit to the data. In addition, among the three profiles, Profile 3 has the most participants with 57.26%, followed by Profile 2 with 30.61%. Profile 1 has the fewest participants with 12.13%.

**Table 4:** Latent profile analysis model fit index.

Model	K	AIC	BIC	ABIC	Entropy	LMR	BLRT
Model 1	14	10013.16	10072.25	10027.81			
Model 2	22	8879.22	8972.08	8902.25	0.90	0.000	0.000
Model 3	30	8344.41	8471.03	8375.81	0.92	0.000	0.000
Model 4	38	8168.51	8329.89	8209.28	0.91	0.049	0.000
Model 5	46	8068.73	8262.87	8116.87	0.92	0.146	0.000

**Figure 3:**
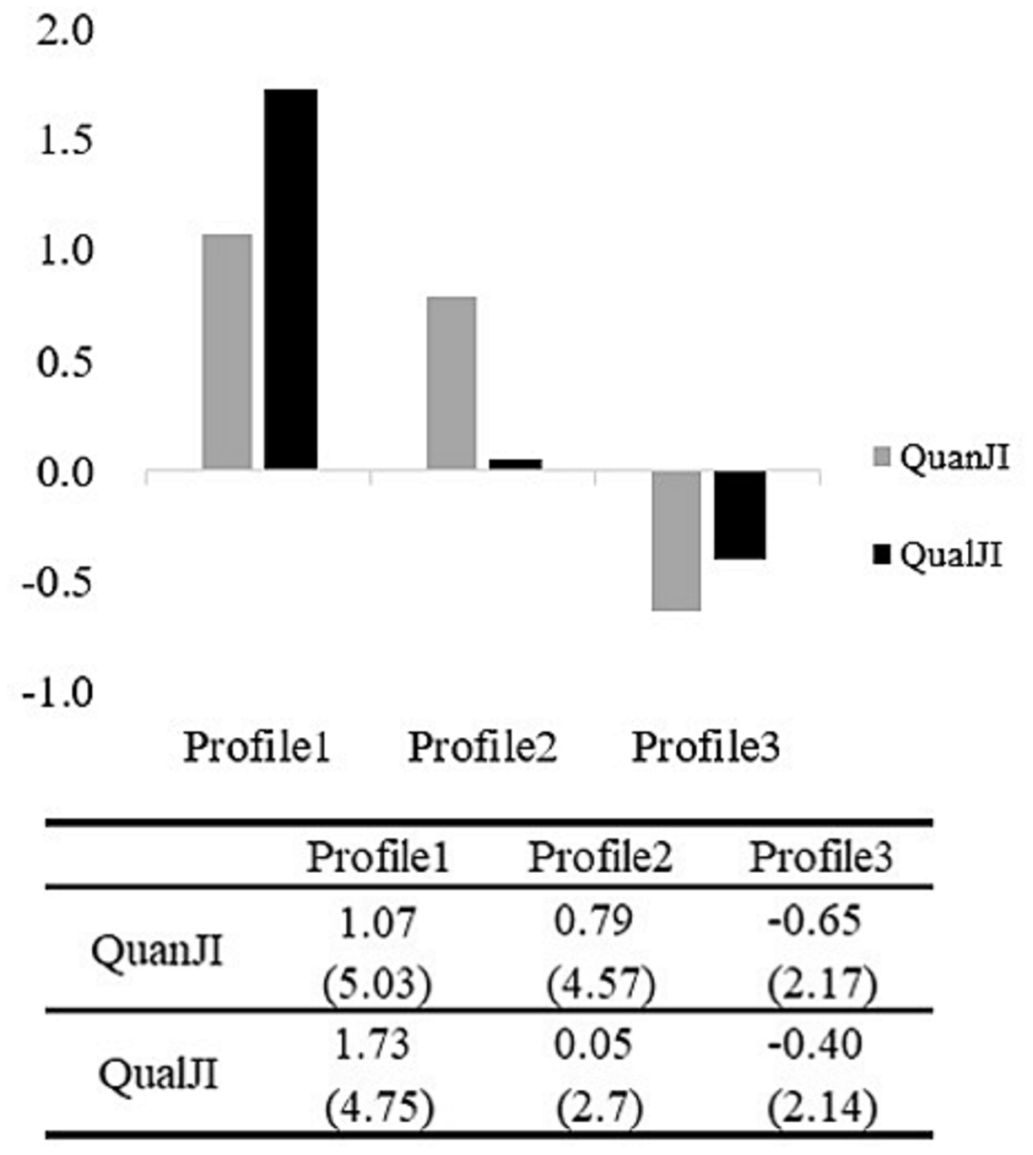
Graphical representation of the profiles. QuanII stands for quantitative job insecurity, and QualJI stands for qualitative job insecurity.

Figure 3 shows graphical representations of the profiles, along with factor and scale scores (in parentheses). The degree of job insecurity decreases from Profile 1 to Profile 3.

Employees in Profile 1 experience high levels of job insecurity, as both their quantitative and qualitative job insecurity scores are higher than the sample mean (quantitative job insecurity factor score: 1.07; qualitative job insecurity factor score: 1.73). Employees in Profile 3 experience low levels of job insecurity, as their quantitative and qualitative job insecurity scores are lower than the sample mean (quantitative job insecurity factor score: −0.65; qualitative job insecurity factor score: −0.40). For employees in Profile 2, a higher quantitative job insecurity score is accompanied by a medium qualitative job insecurity score compared to the sample mean (quantitative job insecurity factor score: 0.79; qualitative job insecurity factor score: 0.05). Based on the above analysis, we label Profile 1 as a balanced profile of high job insecurity, Profile 2 as a profile dominated by quantitative job insecurity, and Profile 3 as a balanced profile of low job insecurity.

#### Differences between profiles in terms of motivation

Univariate analysis revealed significant differences in intrinsic motivation scores between the three profiles (see Table 5). *Post hoc* comparisons revealed that Profile 3 scores were significantly higher than Profile 2 scores (*p* < 0.001), and Profile 2 scores were significantly higher than Profile 1 scores (*p* < 0.001). Impression management motivation scores also differed significantly across the three profiles (see Table 5). *Post hoc* comparisons revealed that Profile 2 and Profile 3 scores were significantly higher than Profile 1 scores (*p* < 0.001), but there was no significant difference between Profile 2 and Profile 3 scores (*p* = 0.86).

**Table 5:** Univariate analyses of the effects of job insecurity profiles.

Outcome	Profile	M ± SD	*F*	*Post hoc* comparisons
Intrinsic motivation	1	3.87 ± 1.31	113.50^**^	Profile 3 > Profile 2 >Profile 1
	2	5.14 ± 1.04		
	3	5.80 ± 0.77		
Impression management motivation	1	4.91 ± 1.09	11.95^**^	Profile 2 > Profile 3 >Profile 1
	2	5.58 ± 0.73		
	3	5.56 ± 1.08		
Proactive innovative behavior	1	4.70 ± 1.18	76.39^***^	Profile 3 > Profile 2 >Profile 1
	2	5.45 ± 0.73		
	3	5.94 ± 0.62		
Reactive innovative behavior	1	4.79 ± 1.09	37.87^***^	Profile 1 > Profile 2 >Profile 3
	2	4.44 ± 0.93		
	3	3.66 ± 1.27		

***p < 0.001, **p < 0.01, *p < 0.05.

#### Differences between profiles in terms of innovative behavior

Further univariate analysis revealed significant differences in both proactive and reactive innovative behavior scores between the three profiles (see Table 5). For proactive innovative behavior, *post hoc* comparisons revealed that Profile 3 scores were significantly higher than Profile 2 scores (*p* < 0.001), and Profile 2 scores were significantly higher than Profile 1 scores (*p* < 0.001). For reactive innovative behavior, *post hoc* comparisons revealed that Profile 1 scores were significantly higher than Profile 2 scores (*p* < 0.05), and Profile 2 scores were significantly higher than Profile 3 scores (*p* < 0.001).

## Discussion

With reference to SDT and from the perspective of job retention motivation, this article examines intrinsic and impression management motivation as mediating variables between quantitative and qualitative job insecurity on the one hand and proactive and reactive innovative behavior on the other, using a variable-centered approach.

The results of this study show that quantitative job insecurity has a significant positive effect on impression management motivation, as hypothesized in H2a. However, contrary to hypothesis H1a, it does not have a significant positive effect on intrinsic motivation. There are two possible explanations for this finding: (1) The relationship between quantitative job insecurity and intrinsic motivation is curvilinear. With low quantitative job insecurity, the work environment is so successful and stable that there is no incentive to change and it is difficult for employees to develop an interest in innovative activities. With high quantitative job insecurity, employees are so concerned about losing their jobs that they shy away from the risks associated with innovative behavior, and intrinsic motivation is suppressed as a result. In contrast, employees with moderate quantitative job insecurity may have relatively high intrinsic motivation to innovate (Zhou and Long, 2011). (2) Quantitative job insecurity has both positive and negative effects on intrinsic motivation at the same time, which cancel each other out. Employees may experience quantitative job insecurity as a disabling or facilitating pressure, leading to a decrease or increase in intrinsic motivation (Zhu and Wu, 2020), which in turn renders the statistical effect insignificant. Furthermore, the results show that, consistent with hypotheses H1b and H2b, qualitative job insecurity has a significant negative effect on both employees’ intrinsic motivation and their impression management motivation. Taken together, this means that while quantitative job insecurity primarily increases employees’ impression management motivation, qualitative job insecurity decreases their intrinsic and impression management motivation.

As hypothesized in H3a, intrinsic motivation showed a significant positive effect on proactive innovative behavior. This is consistent with previous findings, e.g., Montani et al. (2021), who found that employees with high intrinsic motivation analyze problems from different perspectives and try new ways of solving them, while employees with low intrinsic motivation are less curious. In support of H5a, impression management motivation showed a significant positive effect on proactive innovative behavior. Again, this is consistent with previous findings that impression management motivation encourages employees to engage in proactive behaviors such as voice and organizational citizenship (Fuller et al., 2007; Takeuchi et al., 2015). Similarly, Farzaneh and Boyer (2019) found that expected image gains promote proactive innovative behavior. While intrinsic motivation has a significant negative effect on reactive innovative behavior, as hypothesized in H3b, impression management motivation has a significant positive effect on it, as hypothesized in H5b. Employees with low intrinsic motivation are susceptible to external incentives or interference, which increases the likelihood of reactive innovative behavior under organizational pressure. In the case of high impression management motivation, employees will not only engage in proactive innovative behavior to improve their image, but will also engage in reactive innovative behavior because they want to be seen as good employees in light of the rigid innovation goals set by the organization.

Because the effect of quantitative job insecurity on intrinsic motivation (H1a) was not significant, we could only test the mediating role of impression management motivation between quantitative job insecurity and innovative behavior. Consistent with hypothesis H6a, we found that quantitative job insecurity positively influenced proactive and reactive innovative behavior through impression management motivation. In addition, we found evidence for the mediating roles of intrinsic motivation (H4b) and impression management motivation (H6b) between qualitative job insecurity and innovative behavior. Mediated by intrinsic motivation, qualitative job insecurity negatively affects proactive innovative behavior and positively affects reactive innovative behavior. Mediated by impression management motivation, qualitative job insecurity negatively affects proactive and reactive innovative behavior.

As detailed in the mediation analysis above, quantitative and qualitative job insecurity have different effects on employee motivation and innovative behavior. The higher the quantitative job insecurity, the more likely employees are to take actions to demonstrate their value to managers and coworkers in order to secure their jobs (Shoss, 2017). They are more likely to engage in impression management, which promotes proactive and reactive innovative behavior. In the case of high qualitative job insecurity, employees are in a more complex situation: on the one hand, they are pessimistic about their own development prospects in the organization, which leads to a decrease in intrinsic motivation due to the impairment of basic psychological needs and inhibits proactive while encouraging reactive innovative behavior. On the other hand, as the hope for positive development in the current organization decreases, the tendency to look for new employment opportunities outside the organization increases, which in turn leads to a decrease in impression management motivation and consequently inhibits proactive and reactive innovative behavior.

In addition, this study also examined the latent profiles of job insecurity and their impact on motivations and behaviors. The latent profile analysis revealed three profiles of job insecurity: a balanced high job insecurity profile, a profile dominated by high quantitative job insecurity, and a balanced low job insecurity profile. Individuals in the balanced high job insecurity profile are concerned about both job loss and the loss of important job characteristics. Individuals in the profile dominated by high quantitative job insecurity are more concerned about unemployment, but less concerned about the loss of important job characteristics. Finally, individuals in the balanced low job insecurity profile are not concerned about either job loss or the loss of important job characteristics. In our sample, individuals with the balanced low job insecurity profile make up the majority (57.30%). This is consistent with previous studies by Urbanaviciute et al. (2021) and De Cuyper et al. (2019), in which employees with the low insecurity profile also make up the majority (89.20 and 59.80% respectively).

Finally, we found that the three profiles show significant differences in terms of motivation and innovative behavior. Intrinsic motivation is highest for employees in the balanced low job insecurity profile, followed by the profile dominated by high quantitative job insecurity and the balanced high job insecurity profile. Employees in the profile dominated by high quantitative job insecurity show higher impression management motivation than those in the balanced low job insecurity profile, but the difference is not significant. Employees in the balanced high job insecurity profile show significantly lower impression management motivation than employees in the other two profiles. The three job insecurity profiles also differ significantly in terms of proactive and reactive innovative behavior. Employees in the profile dominated by high quantitative job insecurity show intermediate levels of proactive and reactive innovative behavior. Employees with a balanced low job insecurity profile show the highest level of proactive innovative behavior and the lowest level of reactive innovative behavior. This is consistent with the traditional research view that security satisfies employees’ basic needs and creates intrinsic motivation (Vander Elst et al., 2012), which is the source of proactive innovation. Employees with a balanced high job insecurity profile show the lowest level of proactive innovative behavior and the highest level of reactive innovative behavior, which is consistent with Niesen et al.’s (2018) finding that both quantitative and qualitative job insecurity can weaken proactive innovative behavior.

### Theoretical contributions

This study contributes to the literature in three ways: First, it examines the relationship between job insecurity and innovative behavior, including proactive and reactive innovative behavior. While previous research has primarily focused on the effects of job insecurity on employees’ proactive innovative behavior, this study adds new insights to the literature by including reactive innovative behavior as an outcome variable.

Second, we examined the mediating role of motivation in the effect of job insecurity on innovative behavior. While previous research has primarily focused on intrinsic motivation as a mediating variable, this study takes a job preservation perspective and includes impression management motivation as another mediating variable to explain the relationship between job insecurity and innovative behavior.

Third, this study takes a person-centered approach by identifying profiles of job insecurity and examining their effects on motivation and innovative behavior, responding to a call by Debus et al. (2020). This approach provides additional information on the combinations in which quantitative and qualitative job insecurity are experienced by employees and how they affect their motivation and innovative behavior. This compensates for the limitation of the variable-centered approach, which could not capture such heterogeneity within the workforce.

### Practical implications

The results of this study also provide some guidance for organizations facing a dilemma between innovation and workforce management: while employees are needed for innovation, they cannot be guaranteed job stability. Since intrinsic motivation is an important, if not the most important, basis for sustained employee engagement in innovative behavior, qualitative job insecurity must be given special attention, as it can have a significant negative impact on employees’ intrinsic motivation. Organizations should therefore try to take appropriate measures to prevent employees from unjustified fears of losing valued job characteristics (qualitative job insecurity) by establishing transparent and reliable career systems or introducing regular career discussions to build trust and allay employees’ concerns about future career and development prospects.

Knowing that employees use innovative behavior as a means of impression management (Zhao and Zhao, 2019) and thus seek to limit the risk of job loss, organizations can use this to stimulate innovative behavior. When organizational and employee goals are not aligned - the organization needs renewal but employees lack insight or motivation - organizations can take appropriate measures, such as honoring innovation role models or awarding innovation prizes, to extrinsically fuel employees’ reactive innovative behavior by using their impression management motivation to simultaneously increase the organization’s competitiveness and employees’ job security.

### Limitations and directions for future research

This study also has several limitations. First, it is a cross-sectional study. The direction of the causal relationships between job insecurity, motivation, and innovative behavior is based on theoretical considerations and findings. Therefore, it is useful to conduct longitudinal studies in the future to empirically substantiate the causal relationships. Second, innovative behavior is measured based on employee self-report, which may cause common method bias. Future research should use supervisor or peer and employee data for matching. Third, data were collected for all job types, so differences in innovative behavior between workers in different job types were not considered. Job type was only included as a control variable. Considering that different jobs have different requirements for innovative behavior, future research should pay more attention to jobs that require more innovative behavior. Fourth, this study examines the mediating role of intrinsic and impression management motivation between job insecurity and innovative behavior. Future research could also examine additional work motivations, such as achievement motivation or prosocial motivation. Finally, due to the small sample size, only three job insecurity profiles could be identified in this study. In the future, larger samples could be used for analysis to obtain more diverse and detailed profiles.

## Data availability statement

The raw data supporting the conclusions of this article will be made available by the authors, without undue reservation.

## Ethics statement

Ethical review and approval was not required for the study on human participants in accordance with the local legislation and institutional requirements. Written informed consent from the patients/ participants or patients/participants legal guardian/next of kin was not required to participate in this study in accordance with the national legislation and the institutional requirements.

## Author contributions

BM: Conceptualization, Investigation, Methodology, Project administration, Supervision, Writing – review & editing. YZ: Formal analysis, Methodology, Software, Writing – original draft. HL: Writing – review & editing. GM: Conceptualization, Methodology, Project administration, Supervision, Writing – review & editing. RY: Investigation, Methodology, Software, Writing – original draft.
